# Synthesis of Size-Adjustable CsPbBr_3_ Perovskite Quantum Dots for Potential Photoelectric Catalysis Applications

**DOI:** 10.3390/ma17071607

**Published:** 2024-04-01

**Authors:** Hang Li, Jiazhen He, Xiaoqian Wang, Qi Liu, Xuemin Luo, Mingwei Wang, Jinfeng Liu, Chengqi Liu, Yong Liu

**Affiliations:** International School of Materials Science and Engineering (ISMSE), State Key Laboratory of Advanced Technology for Materials Synthesis and Processing, Wuhan University of Technology, Wuhan 430070, China; leehang@whut.edu.cn (H.L.); jiazhenhe0606@163.com (J.H.); 303568@whut.edu.cn (X.W.); liuq@whut.edu.cn (Q.L.); luoxuemin1123@163.com (X.L.); wmw1842591883@163.com (M.W.); liujinf990528@whut.edu.cn (J.L.); liuchengqi42@163.com (C.L.)

**Keywords:** CsPbBr_3_ QDs, quantum confinement effect, blue emission, size-adjustable

## Abstract

As a direct band gap semiconductor, perovskite has the advantages of high carrier mobility, long charge diffusion distance, high defect tolerance and low-cost solution preparation technology. Compared with traditional metal halide perovskites, which regulate energy band and luminescence by changing halogen, perovskite quantum dots (QDs) have a surface effect and quantum confinement effect. Based on the LaMer nucleation growth theory, we have synthesized CsPbBr_3_ QDs with high dimensional homogeneity by creating an environment rich in Br^−^ ions based on the general thermal injection method. Moreover, the size of the quantum dots can be adjusted by simply changing the reaction temperature and the concentration of Br^−^ ions in the system, and the blue emission of strongly confined pure CsPbBr_3_ perovskite is realized. Finally, optical and electrochemical tests suggested that the synthesized quantum dots have the potential to be used in the field of photocatalysis.

## 1. Introduction

Over the past years, metal halide perovskite, a new type of semiconductor material, has attracted wide attention in the photovoltaic [1], luminescence [2] and detection [3] fields because of its excellent photoelectric properties. Compared with traditional semiconductors, perovskites have a special structure, and the general formula is ABX_3_ [4]. This paper focuses on metallic lead halide perovskites with an A-site of MA^+^, FA^+^ or Cs^+^; the B-site is occupied by metal cations such as Pb^2+^ or Sn^2+^, and they have an X-site of Cl^−^, Br^−^ or I^−^. The B-site atoms are in the body center of the cube and form a regular octahedral structure of BX_6_^4−^ with the X-site atoms, the A-site atoms are in the vertex of the cube, and correspondingly, the X-site atoms are in the six face centers of the cube [5,6,7,8,9].

This material is a direct bandgap semiconductor with advantages such as high carrier mobility, long charge diffusion distance, high defect tolerance and a low-cost solution preparation process. Today, perovskite solar cells have achieved a photoelectric conversion efficiency of more than 25.5%, and perovskite quantum dots (QDs) combine the properties of perovskite block materials and traditional semiconductor quantum dots with excellent photoelectric properties, such as high fluorescence quantum yield, extremely narrow half-peak width, adjustable band gap, full-spectrum coverage in visible light, etc. These properties make perovskite quantum dots one of the most promising photoelectric materials at present; they are therefore widely used in light-emitting diodes, solar cells, photodetectors and other fields [10,11,12,13]. Meanwhile, there are relevant examples of the application of quantum dots in the field of photocatalysis [14]. Similarly, composite materials such as perovskite quantum dots combined with other materials to form heterojunctions also have the potential for photocatalytic applications [15]. Quantum dots are quasi-zero-dimensional particles composed of a small number of atoms whose dimensions are close to their Bohr radii [16]. In quantum dots, charge carriers are restricted in all directions and are sometimes referred to as artificial atoms [17]. Quantum dots can be thought of as bridges between molecular clusters and bulk crystals depending on their size. For semiconductors, an extremely important parameter is the gap separating the conduction band (CB) and the valence band (VB), which is fixed for macroscopic crystals and is determined only by the material properties. However, for quantum dots, when the size is reduced to the nanometer level, the electrons are limited by the boundary during movement, and the corresponding energy level will be adjusted to match the change in size. When a quantum dot is excited by a photon of higher energy, the electrons in the VB experience energy fluctuations and are excited to the CB, while a hole is left in the valence band, and the formed electron–hole pair (charge carriers) will be attracted to each other by electrostatic action; the average distance between the electron and the hole is regarded as the Bohr radius (The Bohr radius of CsPbBr_3_ is 7 nm). The diffusion of charge carriers in the semiconductor lattice is related to the Bohr radius. When the size of the quantum dot is less than or close to its Bohr radius, the quantization of energy will be triggered, which will cause the electronic energy-level structure of the semiconductor material to change from a continuous energy level to a discrete energy level, and also cause the band gap between CB and VB to widen; this is the quantum-limited effect [18,19,20]. The quantum confinement effect can also be simply understood: the blue shift of the absorption and fluorescence spectra occurs as the size of the semiconductor decreases, and the blue shift of the spectrum becomes more obvious with a smaller size. In addition, a reduction in size will result in an increase in the proportion of atoms on the surface. Compared with bulk materials, quantum dots have a large specific surface area, and the surface atoms will form a large number of suspension bonds due to incomplete coordination [21,22]. The existence of suspension bonds may introduce defect levels in the semiconductor band gap and cause non-radiative recombination. At this time, the surface plays a decisive role in the structure and optical properties of the quantum dot [23,24,25,26]. In short, there are some properties distinguishing quantum dots compared to bulk materials.

Like traditional quantum dots, perovskite quantum dots can regulate the band gap through the quantum confinement effect to achieve tunable luminescence, and the absorption spectrum will gradually shift toward blue with the decrease in quantum dot size [27,28]. Another, lighter way is to adjust the luminescence through component engineering, that is, by adjusting the proportion of halogens. This is mainly because the CB of perovskite materials is contributed by the 6p orbital of Pb and the np orbital of halogens, and the 6p orbital contribution of Pb is a major part. The VB tops are mainly contributed by the 6s orbits of Pb and the np orbits of halogens [29]. For all metallic lead halide perovskites, the composition of lead is the same, so the band gap of perovskite can be continuously adjusted easily by adjusting the halogen composition, and, thus, the emission of perovskite in the entire visible band can be realized [30]. In addition, due to the soft ionic properties of halide perovskites, there is strong ionic interaction with surface ligands [31,32,33]. This makes it easy to exchange anions between perovskites with different halogens, and it is also easy and convenient to adjust the full spectrum of the visible light band of perovskites through composition engineering [34]. But, unfortunately, it is precisely because of this characteristic that the phase separation of perovskite mixed halogens occurs very easily in practical applications. Conversely, this problem can be fundamentally solved by regulating luminescence through quantum confinement effects. At the same time, how to prepare and control the size of quantum dots and ensure the uniformity of particle size has become a new problem to be solved urgently [35]. Based on previous work, we know that the synthesis of quantum dots follows LaMer nucleation growth theory. It mainly includes three parts; first, the monomer formation stage: because the precursor reacts under certain conditions, the monomer is formed quickly. Second, the nucleation stage: when the concentration of monomer exceeds the critical concentration of nucleation, it will be rapidly nucleated due to supersaturation, and a large amount of monomer will be consumed during nucleation, thereby reducing the monomer concentration to the critical concentration of nucleation again. Third, the growth stage: since the monomer concentration is lower than the critical concentration of nucleation, but still higher than the equilibrium concentration, within this range, QDs will carry out the growth stage [36]. Then, as the concentration of monomers in the solution is depleted, it enters the Ostwald ripening process. This process involves the dissolution of small-size QDs and the continued growth of large-size QDs, which will cause the widening of QDs [37]. The results show that accelerating the monomer formation rate can reduce the nucleation time of QDs, which is conducive to the formation of a more uniform QDs core. The homogeneous core can improve the uniformity of subsequent growth and inhibit the Ostwald ripening process, thus achieving the synthesis of QDs with a narrow size distribution.

Here, we add ZnBr_2_ to create a Br^−^ ion-rich reaction system on the basis of the general hot injection method, and effectively regulate the synthesis of CsPbBr_3_ QDs based on LaMer nucleation growth theory [38]. By simply adjusting the concentration of Br^−^ ions and changing the reaction temperature, CsPbBr_3_ QDs of different sizes with ultra-high particle size uniformity can be obtained easily. These CsPbBr_3_ QDs of similar size exhibit a certain self-assembly behavior due to their high homogeneity, which can be demonstrated by their XRD tests [39]. X-ray photoelectron spectroscopy (XPS) testing can also effectively prove that the Cs, Pb and Br elements in CsPbBr_3_ QDs have a reasonable chemical state environment. In addition, the ultraviolet–visible absorption (UV-vis) spectrum and photoluminescence (PL) spectrum can prove that, based on the quantum confinement effect, the corresponding luminous band of CsPbBr_3_ QDs is significantly blue-shifted with the reduction in their size [40]. Finally, optical and electrochemical tests show that the perovskite quantum dots have great potential for application in the field of photocatalysis.

## 2. Materials and Methods

### 2.1. Materials

Cesium carbonate (Cs_2_CO_3_, Aladdin, Shanghai China, 99.9%), lead bromide (PbBr_2_, Aladdin, Shanghai China, 99.99%), zinc bromide (ZnBr_2_, Aladdin, Shanghai China, 99%), oleic acid (OA, Aladdin, Shanghai China, AR), oleylamine (OAm, Aladdin, Shanghai China, 80~90%), 1-octadecene (ODE, Aladdin, Shanghai China, >90% (GC)), n-hexane (Aladdin, Shanghai China, >99%), acetone (Aladdin, Shanghai China, >99%).

### 2.2. Methods

#### 2.2.1. Preparation of CsOA Precursors

The Cs-oleate precursor solution was prepared in a 50 mL three-necked round-bottomed flask by dissolving Cs_2_CO_3_ (0.4 g) in a mixture of oleic acid (OA, 5 mL) and 1-octadecene (ODE, 15 mL). After drying with a vacuum pump for 1 h at 120 °C, it was heated under N_2_ protection to 140 °C until all Ca_2_CO_3_ dissolved to form Cs-oleate. The Cs-oleate precursor solution was preheated to 120 °C before injection.

#### 2.2.2. Synthesis of CsPbBr_3_ Quantum Dots (QDs)

ODE (10 mL), PbBr_2_ (0.138 g) and ZnBr_2_ were loaded in a 50 mL 3-necked flask and vacuum-dried for 1 h at 120 °C, and then oleylamine (2 mL) and OA (2 mL) were injected under a N_2_ atmosphere. After the PbBr_2_ and ZnBr_2_ dissolved, the temperature was set to a different temperature, and the Cs-oleate solution (0.8 mL, 0.125 M in ODE) was quickly injected in. The reaction mixture was cooled by an ice-water bath after reacting for 30 s for the crystal growth. The product was collected by centrifugation. The amount of ZnBr_2_ added depended on the molar ratio of bromine/lead. When the molar ratio of Br:Pb = 6:1, ZnBr_2_ (0.17 g) was required, and when the molar ratio of Br:Pb = 10:1, twice as much was required.

#### 2.2.3. Purification of CsPbBr_3_ Quantum Dots (QDs)

For the product in the centrifugation, the supernatant was left on the bench top under ambient conditions for ~3 h until the unreacted salts precipitated. Then the mixture was centrifuged to get the clear supernatant. With a proper amount of the clarified solution, ~5 mL of acetone was slowly added until the mixture became turbid to avoid decomposition of the QDs. Then the QDs were centrifuged at 10,000 rpm for 10 min and the precipitate was collected and dissolved in ~500 μL of hexane.

### 2.3. Characterization

#### 2.3.1. Characterization via Transmission Electron Microscopy (TEM)

Images captured via transmission electron microscopy (TEM) were generated utilizing a JEOL JEM-1400 Plus microscope (Beijing, China). This instrument, featuring a thermionic emission source, was set to function at an acceleration power of 120 kV. For the examination, quantum dot (QD) specimens underwent a precise preparation process, involving the deposition of a thinned QD mixture in n-hexane onto grids coated with carbon on copper. Following this, the analysis of the QDs’ size distribution was conducted through the application of Nano Measurer software (1.2), bearing version number 1.02.0005.

#### 2.3.2. Characterization via X-ray Diffraction (XRD)

X-ray diffraction (XRD) measurements were conducted utilizing a Bruker D8 ADVANCE diffractometer, from Karlsruhe, Germany, employing Cu-Kα radiation at a wavelength of 1.540598 Å. Data collection covered a 2θ range from 10° to 60°, utilizing a step increment of 0.05° and a dwell time of 0.5 s per step. The instrument was set to a voltage of 40 kV and a current of 40 mA. To prepare for XRD, a dense suspension of quantum dots (QDs) in n-hexane was drop-cast onto silicon dioxide/silicon (SiO_2_/Si) supports. Analysis of the XRD patterns was subsequently performed with Jade 6.5 software.

#### 2.3.3. X-ray Photoelectron Spectroscopy (XPS) Characterization

Spectra from X-ray photoelectron spectroscopy (XPS) of perovskite quantum dots were captured with an AXIS SUPRA spectrometer from Kratos Analytical, part of the Shimadzu Group, located in Manchester, UK. This device features a Thermo Scientific Kα spectrometer and utilizes a monochromatic Al-Kα X-ray for illumination. Data collected were then transformed into VGD format and analyzed using Avantage software (599-31), release 5.9922. Calibration of the binding energy scale was aligned using the C 1 s peak, attributing a binding energy of 284.8 eV specifically to the carbon–carbon (C–C) bond.

#### 2.3.4. Fluorescence Spectrum Characterization

Optical absorption spectra in the UV–visible range were obtained using a Shimadzu UV-1800 spectrophotometer (Kyoto, Japan), which was operated with UVProbe 2.52 software. Concurrently, the emission characteristics were assessed through steady-state photoluminescence (PL) spectra, recorded using a Shimadzu RF-6000 spectrophotometer (Kyoto, Japan) equipped with LabSolutions RF software (1.11). For CsPbBr_3_, the excitation wavelength (λ_ex_) utilized was 400 nm. The preparation for optical evaluation involved the dilution of quantum dot (QD) solutions in n-hexane, followed by their allocation into quartz cuvettes, each with a path length of 10 mm, to ensure accurate measurements.

#### 2.3.5. Electrochemistry (Photo) Measurements

Photocurrent and electrochemical impedance analyses on halide perovskite quantum dots (QDs) were conducted using a Zennium workstation, supplied by Zahner Company of Kronach, Germany. This investigation utilized a tri-electrode configuration comprising a sample-based working electrode, a counter-electrode of platinum disk, and a Ag/AgCl reference electrode (in saturated KCl solution) with a potential of +0.1989 V relative to the Normal Hydrogen Electrode (NHE) within an electrolyte solution of 0.5 M Na_2_SO_4_. Illumination for the photocurrent assessments was provided by a 405 nm wavelength LED, with its intensity quantified using a Newport photometer (Irvine, CA, USA). The electrochemical impedance spectra were acquired at a potential of −0.1 V versus NHE, with application of a 10 mV signal over a frequency range from 100 mHz to 20 kHz, in a 0.5 M Na_2_SO_4_ solution.

## 3. Results

**Scheme 1:**
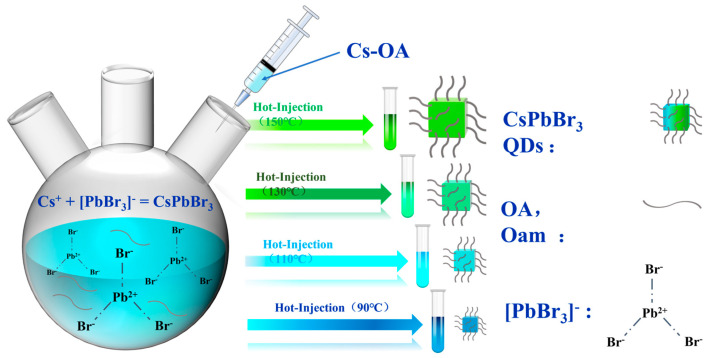
Illustrative representation of the CsPbBr_3_ QDs synthesis process.

The traditional synthesis of CsPbBr_3_ nanocrystals involves simple dissolution of PbBr_2_ and ligands in a solvent and hot injection of cesium oleate to obtain the corresponding halogen perovskite nanocrystals. Although monodisperse nanocrystals have been synthesized successfully, their sizes are not uniform, and effective size control methods are lacking. Based on the LaMer nucleation growth theory, it can be seen that this is an extremely rapid explosive nucleation process, accompanied by the Oswald maturation process in parallel with crystal growth. Therefore, a BR^−^-rich reaction system was constructed by introducing ZnBr_2_, which can improve the formation of perovskite monomer and shorten the nucleation time. The homogeneous core is conducive to inhibiting the Oswald ripening process in the later stage of growth, avoiding size widening and obtaining a narrow size distribution of quantum dots. In addition, the size of the perovskite QDs can be adjusted by changing the reaction temperature during hot injection. As the size of QDs decreases, we obtain strongly confined CsPbBr_3_ QDs. Due to the great quantum confinement effect, the luminous wavelength of CsPbBr_3_ QDs shifts from green to blue. Compared with the component engineering of Cl/Br mixed halogens, the blue light formed based on dimension engineering fundamentally solves the problem of easy phase separation of mixed halogens. At the same time, with the addition of excessive Br^−^ ions, the Br vacancy defects on the surface of perovskite are greatly passivated [41]. In bromine-rich reaction systems, Pb^2+^ and Br^−^ ions are combined to form a complex of [PbBr_3_]^−^ firstly, and then the complex is transformed into an octahedron. Once the octahedron is formed, sufficient nucleation sites are provided for rapid nucleation with the addition of Cs-OA. We obtained CsPbBr_3_ QDs of different sizes at different reaction temperatures (150 °C, 130 °C, 110 °C, 90 °C) (Scheme 1). Based on the weak-to-strong quantum confinement effect, the continuously tunable emission of CsPbBr_3_ QDs from green light to blue light was realized.

**Figure 1:**
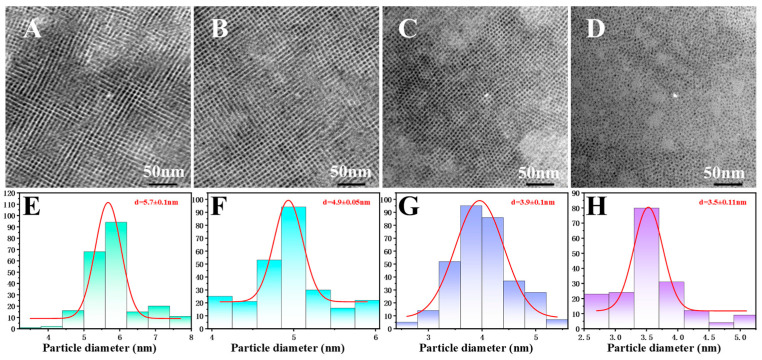
(**A**–**D**) TEM images of CsPbBr_3_ QDs at different reaction temperatures, respectively (scale bar: 50 nm) and (**E**–**H**) their size-of-particle-diameter distribution diagrams.

Shown in Figure 1A–D are TEM images of CsPbBr_3_ QDs corresponding to reaction temperatures of 150 °C, 130 °C, 110 °C and 90 °C, respectively. The size of CsPbBr_3_ QDs can be visually observed through TEM images. It can be clearly seen from the TEM images that the size of the obtained CsPbBr_3_ QDs decreased with the gradual decrease in the reaction temperature during hot injection. In addition, we calculated the sizes of hundreds of quantum dots, made a bar chart, and placed it under the TEM images of quantum dots we obtained, arranged by the corresponding reaction temperatures (Figure 1E–H). According to the information in the bar chart, the average size and particle size distribution of CsPbBr_3_ QDs obtained, from high to low reaction temperature, are d = 5.7 ± 0.1 nm, d = 4.9 ± 0.05 nm, d = 3.9 ± 0.1 nm and d = 3.5±0.11 nm, respectively. In addition, comparing Figure S1 and Figure 1, it can be found that as the molar ratio of bromine to lead increases from Br: Pb = 6:1 to Br: Pb = 10:1, the sizes of QDs in the system with higher concentrations of Br^−^ ions at the same reaction temperature become smaller. This shows that in addition to the temperature condition, the change in Br^−^ ion content in the system also really affects the size of the quantum dot. At the same time, in an environment with high-energy particle impacts such as TEM, there are no white spots, which indicates the destruction of the structure, and small black patches, which indicate the collapse of the structure, leading to the precipitation of PbBr_2_. Studies indicate that increasing the concentration of halogen ions can improve the radiation resistance of halide perovskite QDs [42]. This may be attributed to the fact that with the increase in Br^−^ ion content in the system, more nucleation sites are formed while the surface defects are passivated. The increase in nucleation sites greatly accelerates the nucleation process and, thus, effectively inhibits Oswald ripening in some aspects, which means that the sizes of the perovskite quantum dots with extremely high size uniformity obtained by the improved method in this paper can be controlled simply and effectively. What is more interesting is that, compared with the monodisperse CsPbBr_3_ QDs synthesized by the general method, they show a chaotic arrangement (Figure S2). The improved CsPbBr_3_ QDs synthesized in this paper are arranged in an orderly manner on the substrate, and show a trend of self-assembly. It should be clear that this trend of self-assembly has high requirements for the dimensional uniformity of the assembled units, which is also evidence that the synthesized CsPbBr_3_ QDs have ultra-high dimensional uniformity. This high dimensional uniformity and size adjustability will also greatly facilitate the installation of CsPbBr_3_ QDs in channel materials, further developing their potential in areas such as catalysis.

**Figure 2:**
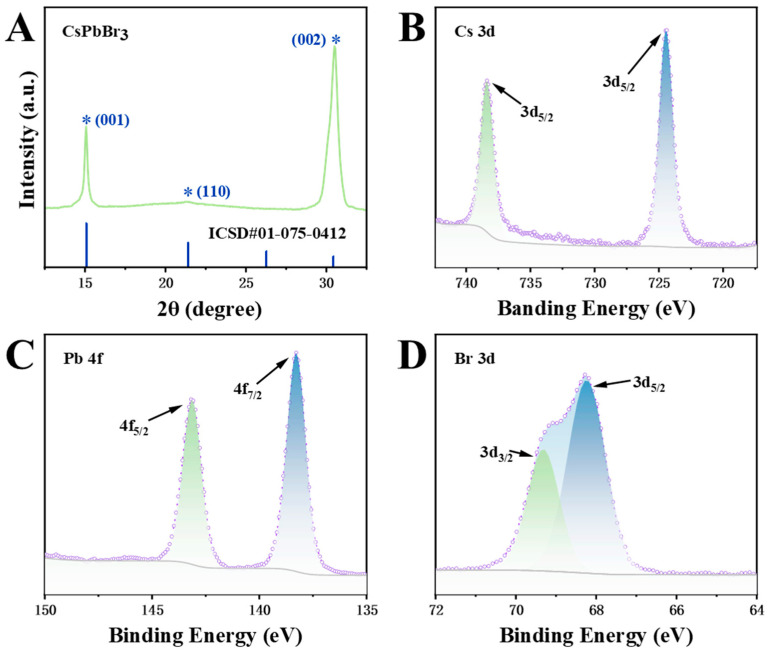
(**A**) XRD pattern and (**B**–**D**) XPS spectra of CsPbBr_3_ QDs.

It is noteworthy that the X-ray diffraction (XRD) pattern exhibits only two highly intense peaks, near the diffraction angles 2θ = 15° and 2θ = 30°. These peaks correspond to the (001) and (002) crystal faces of CsPbBr_3_ perovskite, respectively. The presence of these specific peaks, in accordance with the ICSD code 029073, confirms the cubic crystal structure of CsPbBr_3_ QDs (Figure S3). Just like the extremely ordered arrangement of quantum dots in the TEM images, CsPbBr_3_ QDs are arranged along the preferred orientation of the (001) crystal plane, with structural coherence, so that there are extremely strong diffraction peaks at the peaks of the (001) crystal plane and the (002) crystal plane in the XRD pattern. The special XRD pattern of the synthesized CsPbBr_3_ QDs is in good agreement with the special arrangement in the TEM images. Figure 2B–D and Figure S4 show the XPS spectrum of CsPbBr_3_ QDs. According to the curve of the spectrum, the chemical valence states of Cs, Pb and Br were analyzed [43]. Figure 2B shows the binding energy curve of the Cs 3d orbit, where the peaks corresponding to Cs 3d_5/2_ and Cs 3d_3/2_ are located near 724 eV and 738 eV, respectively. Similarly, Figure 2C shows the binding energy curve of the Pb 4f orbit, where the peaks corresponding to Pb 4f_7/2_ and Pb 4f_5/2_ are located near 138 eV and 143 eV, respectively. In addition, in Figure 2D, the coupling peaks near 68 eV and 69 eV come from the Br 3d orbit, representing Br 3d_5/2_ and Br 3d_3/2_, respectively. In the above peak location, the peak location of the Pb 4f track is similar to that recorded in reference [44].

**Figure 3:**
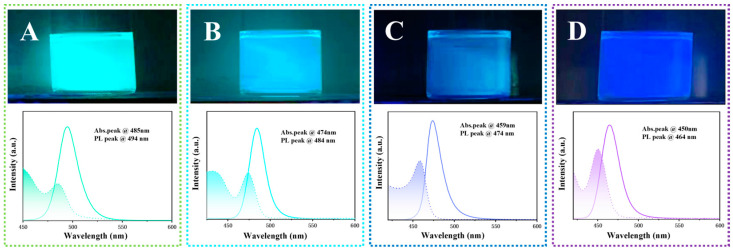
(**A**–**D**) images of CsPbBr_3_ QDs at different reaction temperatures under ultraviolet lamp excitation and the corresponding UV-vis and PL spectra.

Subsequently, we performed optical characterization of CsPbBr_3_ quantum dot samples at various reaction temperatures, including UV-vis and photoluminescence (PL) spectra. As the average particle size of CsPbBr_3_ QDs decreases, the quantum confinement effect changes from weak to strong, the continuous electronic energy levels split into discrete energy levels, and the energy levels are constantly widened. Shown in Figure 3A–D are CsPbBr_3_ quantum dot samples corresponding to reaction temperatures of 150 °C, 130 °C, 110 °C and 90 °C, respectively. We dispersed them in hexane and obtained the transition of excitation light from green to blue light under the excitation of an ultraviolet lamp. The corresponding UV-vis absorption peak and PL emission peak also gradually transited from a green band to a deep blue band, indicating that the band gap gradually widened. According to the detailed information shown in the UV-vis spectra and PL spectra in Figure 3, the emission peak positions of PL shift from 494 nm, 484 nm and 474 nm to 464 nm (deep blue light), while the corresponding absorption peak positions shift from 485 nm, 474 nm and 459 nm to 450 nm. It should be noted that the intrinsic PL excitation band of CsPbBr_3_ QDs should be around 520 nm. For details, see the illustration in Figure S5. The solution of CsPbBr_3_ QDs dispersed with larger sizes appears bright green under ultraviolet irradiation. The excited light color of the CsPbBr_3_ QDs solution obtained at the reaction temperature of 150 °C under the UV lamp is more like the transition color between green and blue, because the average particle size of the sample at this time was d ≈ 5.7 nm < 7 nm. At this time, an obvious quantum confinement effect appeared, and the original continuous electronic energy levels split into discrete energy levels. As the energy level widened, the band gap increased and the PL peak position shifted to blue. The quantum confinement effect can also increase the exciton binding energy of the crystal; the exciton binding energy in the halogen perovskite film is about 25 meV, and the excitons in the perovskite film will decompose into free electrons and holes at room temperature. For perovskite quantum dots, the exciton binding energy can reach hundreds of meV, and the increase in exciton binding energy increases the possibility of radiation recombination, which can significantly improve luminous performance. In addition, the adjustable band gap of CsPbBr_3_ QDs also has great potential in the field of photocatalysis. By adjusting the band gap of CsPbBr_3_ QDs and other materials to form heterostructures (Figure S7), it is easier to form a type II band structure, effectively realize the separation of electrons and holes, and improve the catalytic performance.

**Figure 4:**
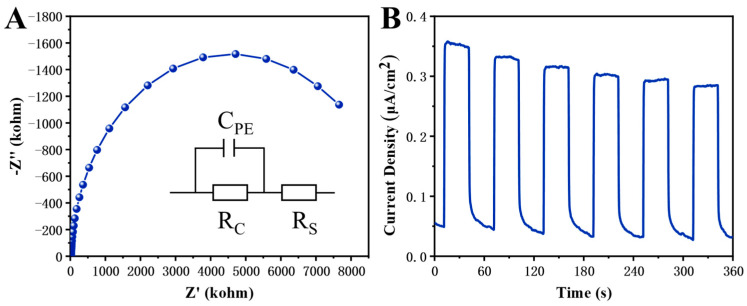
(**A**) Electrochemical impedance spectroscopy (Nyquist plot) of CsPbBr_3_ QDs synthesized at a reaction temperature of 150 °C. The inset illustrates the equivalent circuit model. (**B**) Instantaneous photocurrent response of CsPbBr_3_ QDs in neutral water (0.5 M Na_2_SO_4_) at −0.1 V vs. NHE.

CsPbBr_3_ quantum dots (QDs), fabricated at a temperature of 150 °C, were subjected to assessments of photocurrent response and electrochemical impedance spectroscopy (EIS), as illustrated in Figure 4. The equivalent circuit analysis involved C_PE_ as the notation for double-layer capacitance, with R_S_ representing the resistance across the electrolyte between the reference and working electrodes, and R_C_ indicating the resistance related to charge transfer at the electrode interface. The EIS patterns for the CsPbBr_3_ QDs showcased a distinct semicircle, a marker for charge transfer resistance. To accurately measure the photocurrent response exclusive of external effects, measurements were systematically taken with the initiation of the light source. Remarkably, when the illumination was discontinued, the photocurrent density approached zero, but surged immediately when the light was reinstated after a 30 s interval. Such periodic fluctuation in current, with light modulation every 30 s, underscores the CsPbBr_3_ perovskite’s adeptness at carrier transport. This efficiency in carrier migration underscores the potential of CsPbBr_3_ QDs for future photoelectrocatalysis applications.

## 4. Conclusions

Based on the synthesis of perovskite by the hot injection method, we introduced more Br^−^ ions by adding ZnBr_2_ to create a bromine-rich reaction environment to regulate the nucleation and growth process of perovskite, and finally synthesized monodisperse, extremely uniform and adjustable CsPbBr_3_ QDs. Due to the presence of excessive Br^−^ ions in the solution, the reaction system had abundant nucleation sites, which greatly sped up the explosive nucleation process in the hot injection stage, and effectively inhibited the Oswald ripening process in the subsequent crystal growth. Thus, CsPbBr_3_ QDs with an extremely uniform size were obtained. We can regulate the size of CsPbBr_3_ QDs by adjusting the reaction temperature during hot injection and the amount of Br^−^ ions added. The general rule is as follows: when the content of Br^−^ ions in the system is constant, the size of the obtained quantum dots becomes smaller and smaller as the reaction temperature gradually decreases. Similarly, when the temperature is constant, the size of the quantum dot is negatively correlated with the Br^−^ ion content in the system to a certain extent. In addition, because the size is uniform enough, there is an interesting self-assembly arrangement, which can be clearly seen in TEM images and is to some extent supported by XRD. In addition, based on the quantum confinement effect, the size changes of CsPbBr_3_ QDs also bring special optical properties. When the size of the quantum dot enters the critical value of the Boer exciton radius (7 nm), with a further reduction in the average size, its photoluminescence wavelength shifts to a deep blue band. The results of spectral and structural characterization show that the emission of CsPbBr_3_ perovskite, from green light to blue light, can be continuously adjusted by adjusting the sizes of quantum dots. In general, on the one hand, from the perspective of structure, CsPbBr_3_ QDs can achieve more efficient catalytic performance by attaching to other materials, and the adjustable size property greatly facilitates this method. Furthermore, based on the change in band gap width caused by the size effect, the formation of a type II band structure with other materials also has the potential to be applied in the field of catalysis.

## Data Availability

Data are available in a publicly accessible repository that does not issue DOIs or upon request from the corresponding author.

## Supplementary Materials

The following supporting information can be downloaded at: https://www.mdpi.com/article/10.3390/ma17071607/s1, Figure S1: (A–D) TEM images of CsPbBr_3_ QDs at different reaction temperatures (scale bar: 50 nm) and (E,F) their size-of-particle-diameter distribution diagrams; Figure S2: Image of CsPbBr_3_ QDs at different reaction temperatures under ultraviolet lamp excitation and the corresponding UV-vis and PL spectra; Figure S3: (a) HR-TEM image and its magnified local view; (b) the reciprocal space image after FFT; (c) the lattice image obtained by inverse FFT and the reciprocal space image; Figure S4: The XPS spectrum of CsPbBr_3_ QDs; Figure S5: Image of CsPbBr_3_ QDs under ultraviolet lamp excitation and the corresponding PL spectra; Figure S6: (a) TEM image and (b) EDS spectra of CsPbBr_3_ QDs; Figure S7: The relationship between absorbance and photon energy. The band gap (B_g_) of CsPbBr_3_ QDs of different sizes.
