# Supplementary material for: Synthesis of Size-Adjustable CsPbBr3 Perovskite Quantum Dots for Potential Photoelectric Catalysis Applications
Source: Materials (Basel). 2024 Apr 1;17(7):1607. doi: 10.3390/ma17071607 (PMC11012633; doi:10.3390/ma17071607)
Supplement: Supplementary file 1 [file materials-17-01607-s001.zip › materials-2936377-supplementary.pdf]

*Article*

# Synthesis of Size-Adjustable $CsPbBr_3$ Perovskite Quantum Dots for Potential Photoelectric Catalysis Applications

**Hang Li, Jiazhen He, Xiaoqian Wang, Qi Liu, Xuemin Luo, Mingwei Wang, Jinfeng Liu, Chengqi Liu and Yong Liu ***

International School of Materials Science and Engineering (ISMSE), State Key Laboratory of Advanced Technology for Materials Synthesis and Processing, Wuhan University of Technology, Wuhan 430070, China; leehang@whut.edu.cn (H.L.); jiazhenhe0606@163.com (J.H.); 303568@whut.edu.cn (X.W.); liuq@whut.edu.cn (Q.L.); luoxuemin1123@163.com (X.L.); wmw1842591883@163.com (M.W.); liujinf990528@whut.edu.cn (J.L.); liuchengqi42@163.com (C.L.)

* Correspondence: liuyong3873@whut.edu.cn

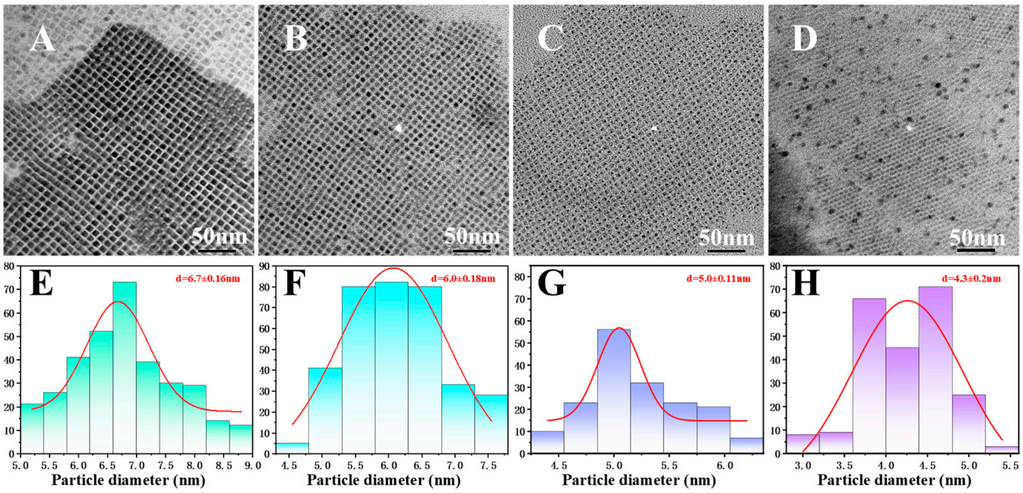


**Figure S1. (A-D)** TEM images of $CsPbBr_3$ QDs at different reaction temperatures(scale bar: 50nm) and **(E-F)** their size of particle diameter distribution diagrams.

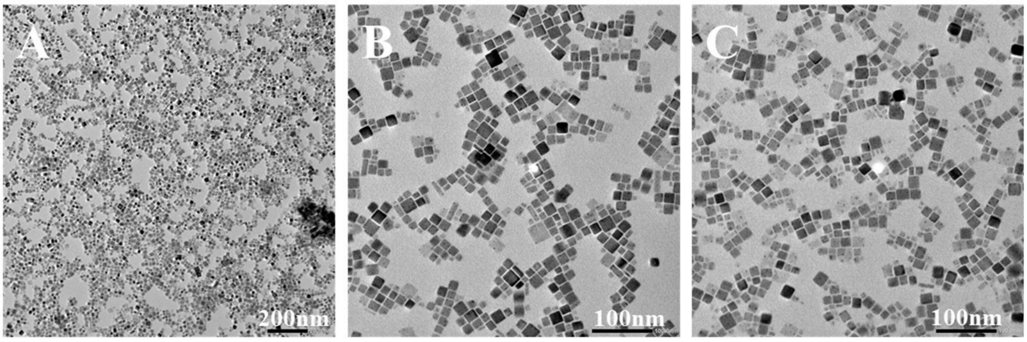


**Figure S2.** image of $CsPbBr_3$ QDs at different reaction temperatures under ultraviolet lamp excitation and its corresponding UV-vis and PL spectra.

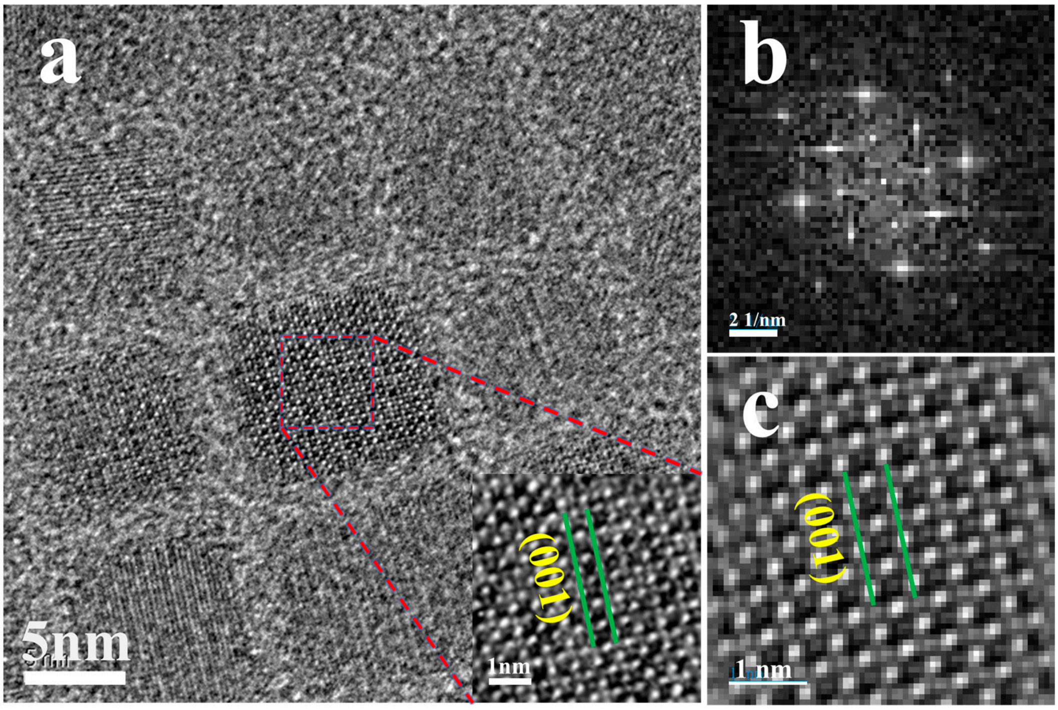


**Figure S3. (a)** HR-TEM image and its magnified local view; **(b)** The reciprocal space image after FFT; **(c)** The lattice image obtained by inverse FFT the reciprocal space image.

As shown in **Figure S3**, the reciprocal space image obtained from the Fast Fourier Transform (FFT) of the High-Resolution TEM (HR-TEM) image of the perovskite quantum dots displays a lattice type that aligns well with the XRD phase. Additionally, we measured the interplanar spacing of the (100) crystal plane, which essentially aligns with the description on the ICSD card (0.587nm).

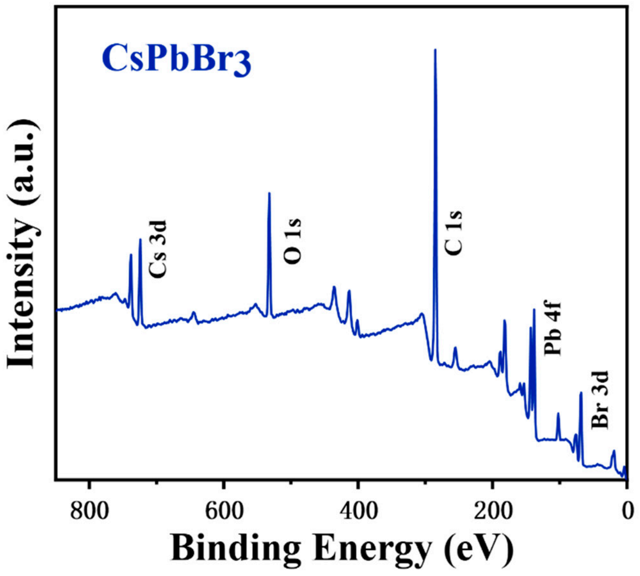


**Figure S4.** The XPS spectrum of $CsPbBr_3$ QDs.

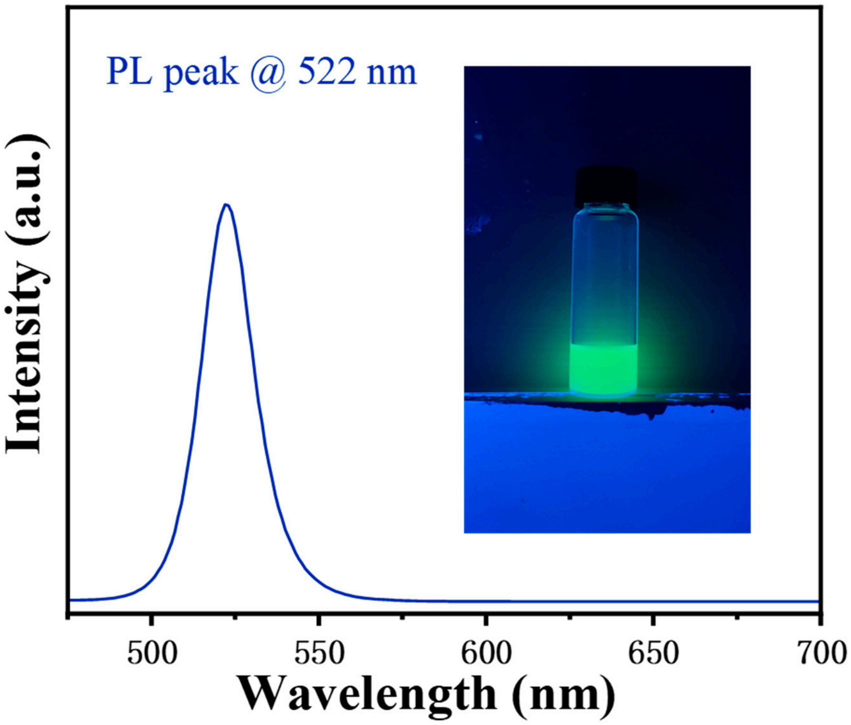


**Figure S5.** image of $CsPbBr_3$ QDs under ultraviolet lamp excitation and its corresponding PL spectra.

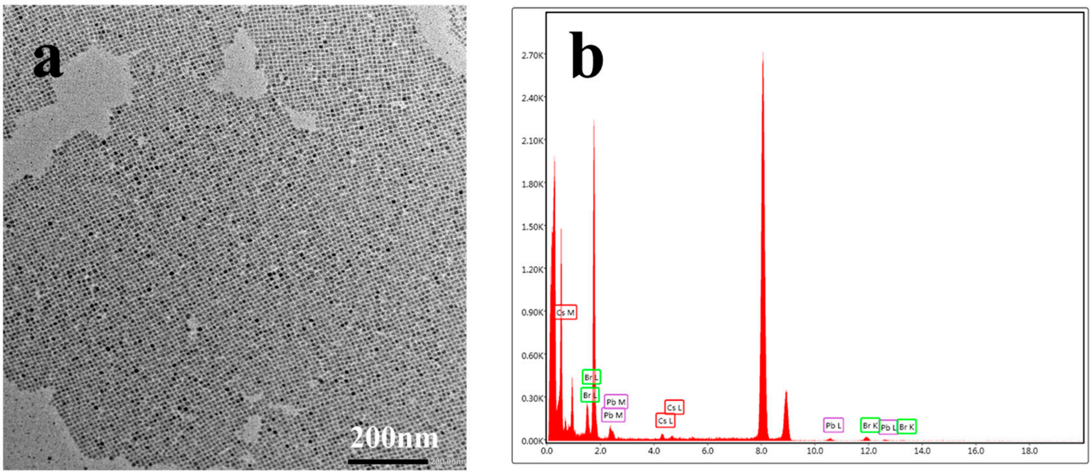


**Figure S6.** **(a)** TEM image and **(b)** EDS spectra of $CsPbBr_3$ QDs.

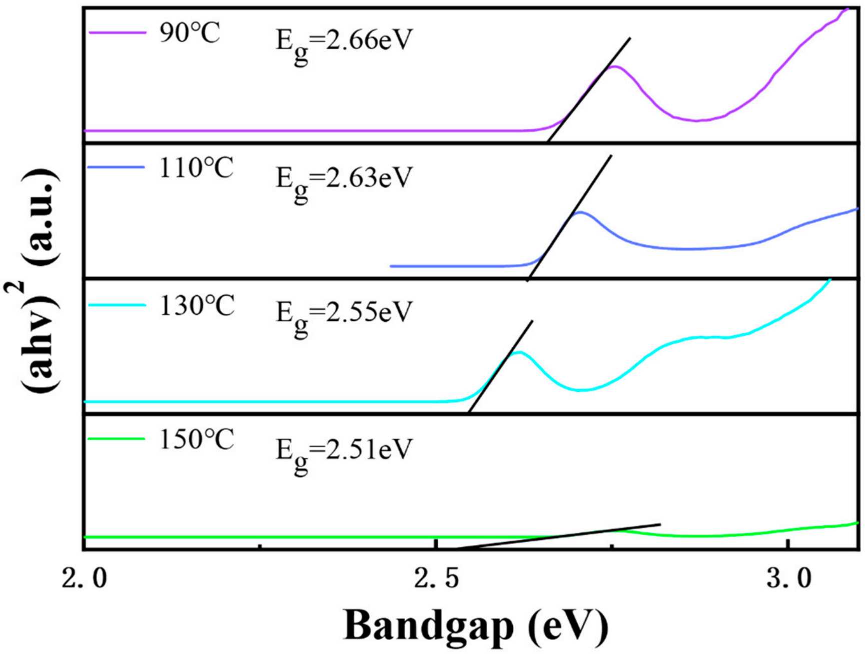


**Figure S7.** The relationship between absorbance and photon energy. The band gap ($B_g$) of $CsPbBr_3$ QDs of different sizes.

As illustrated in **Figure S7**, a decrease in temperature results in a reduction in the size of the quantum dots. This reduction enhances the quantum confinement effect, which in turn leads to an increase in the corresponding band gap width.
